# Combined Evaluation of mRNA and Protein Expression, Promoter Methylation, and Immune Infiltration of UBE2I in Pan-Digestive System Tumors

**DOI:** 10.1155/2022/1129062

**Published:** 2022-09-20

**Authors:** Shuai Huang, Xiangkun Wang, Kai Luo, Xudong Zhang, Zhongyuan Liu, Renfeng Li

**Affiliations:** Department of Hepatobiliary and Pancreatic Surgery, The First Affiliated Hospital of Zhengzhou University, Zhengzhou, 450052 Henan, China

## Abstract

**Background:**

Digestive system tumors (DSTs) have high morbidity and mortality worldwide. This study explored the potential value of ubiquitin-conjugating enzyme E2 I (*UBE2I*) in pan-digestive system tumors (pan-DSTs).

**Methods:**

Differential expression, tumor stages, and survival outcomes of *UBE2I* in pan-DSTs were determined using the GEPIA database. The TIMER database was used to confirm the correlation of *UBE2I* expression with pan-DSTs and immune infiltrates. Differential analyses of *UBE2I* promoter methylation and protein levels were performed using the UALCAN database. The underlying mechanisms of *UBE2I* involvement in pan-DSTs were visualized using interaction networks. The diagnostic value of *UBE2I* in pan-DSTs was identified using the Oncomine database.

**Results:**

*UBE2I* was differentially and highly expressed in cholangiocarcinoma (CHOL), pancreatic adenocarcinoma (PAAD), colon adenocarcinoma (COAD), rectal adenocarcinoma (READ), liver hepatocellular carcinoma (LIHC), and stomach adenocarcinoma (STAD). According to survival analysis, upregulated *UBE2I* was associated with adverse overall and disease-free survival in PAAD and favorable overall survival in READ. *UBE2I* expression was partially linked to the purity of immune infiltration in COAD, LIHC, PAAD, READ, and STAD, as indicated by the immune infiltration analysis. Promoter methylation analysis showed differential and high methylation of *UBE2I* in PAAD as well as stratified analysis by gender, nodal metastasis, and race. Protein expression analysis in colon cancer revealed that *UBE2I* had differential and high expression in tumors as well as stratified analysis by gender, tumor histology, race, and tumor stage. Mechanism explorations demonstrated that in COAD and PAAD, *UBE2I* was involved in spliceosomal snRNP complex, Notch signaling pathway, etc. Diagnostic analysis indicated that *UBE2I* had consistent diagnostic value for COAD and PAAD.

**Conclusions:**

Upregulated *UBE2I* may be a diagnostic and surveillance predictive signature for PAAD and COAD. The potential significance of immune infiltrates and promoter methylation in PAAD and COAD needs further exploration.

## 1. Introduction

The term digestive system tumor (DST) describes a group of tumors that affect diverse digestive system tissues, involving esophagus, stomach, liver, pancreas, colon, and rectum [1]. The majority of these neoplasms are carcinomas (>90%) [2]. DST remains a leading cause of tumor-related mortality, causing approximately three million deaths worldwide each year [3, 4]. In recent years, the number of DST cases has significantly increased, highlighting the urgent requirement for more effective treatment strategies [5]. Despite significant advances in molecular medicine in disease prevention, diagnosis, and treatment, the prognosis of DSTs remains poor due to their increasing prevalence, diagnosis at advanced stages, tumor recurrence, and drug resistance [6]. Identification of novel molecular targets for DSTs may therefore provide insights into the development of effective therapeutic drugs.

SUMOylation is a reversible protein posttranslational modification process in which small ubiquitin-like modifier (SUMO) proteins are covalently bound to target proteins' lysine residues [7]. The SUMO system modulates a wide range of cellular processes, including cell division, chromatin segregation, transcription, signal transduction, protein stability, and translocation [7]. Ubiquitin conjugating enzyme E2 I (*UBE2I*) is a crucial component of this system, augmenting the ubiquitination and proteasomal flux of target proteins [7]. SUMOylation is an important posttranslational modification that fine-tunes almost all cellular functions and pathological processes, playing an important role in human tumorigenesis [8]. The SUMO pathway can induce cell proliferation, antiapoptosis, and metastatic potential by regulating proteins involved in carcinogenesis [9–13].

siRNA-mediated suppression of *UBE2I* is reported to inhibit LC3-II, an autophagy marker protein, and conversely promote the expression of SQSTM1/p62, which translocates ubiquitinated proteins to the proteasome and the autophagosome precursor—phagophore [7]. Furthermore, increased SUMOylation exerts a cardioprotective effect and decreases morbidity in proteotoxic cardiac disease [7]. *UBE2I* was significantly downregulated in patients with chromosome 9 open reading frame 72 and neurological progranulin mutations as well as sporadic frontotemporal dementia and age-matched controls [14]. Knockdown of *UBE2I*, also known as *UBC9*, impairs Notch 1-activated breast epithelial cell proliferation, indicating the potential value of *UBE2I* in targeted treatment of Notch-driven breast cancer [15]. In addition, differentially expressed *UBE2I* was observed in all four (clear cell, endometrioid, mucinous, and serous) subtypes of epithelial ovarian cancer [16]. Another study by Poleshko et al. [17] demonstrated that enzymes of the SUMO pathway are critical for the maintenance of epigenetic silencing. Furthermore, *UBE2I* upregulation was reported to be linked to disease development in a mouse model of necrotizing enterocolitis [18]. However, limited knowledge is available regarding the expression patterns and functions of *UBE2I* in digestive disorders, in particular, DSTs. Accordingly, the motivation and novelty of the study is to investigate the potential roles played by *UBE2I* and its underlying mechanism in pan-DSTs.

## 2. Materials and Methods

### 2.1. *UBE2I* Expression Patterns in Different Types of Cancers and Normal Tissue Specimens

*UBE2I* mRNA levels in pan-cancerous and normal tissue specimens from the UALCAN database (http://ualcan.path.uab.edu/) [19], a comprehensive, user-friendly, and interactive web resource for cancer omics data analyses, were examined. Then, the Gene Expression Profiling Interactive Analysis (GEPIA; URL: http://gepia2.cancer-pku.cn/#index) [20], a newly developed server for RNA sequencing expression data analyses of 9736 carcinoma tissues and 8587 normal counterparts from the TCGA and GTEx projects, was utilized to determine differential expression patterns of *UBE2I* in pan-DSTs using standard processing pipelines. Correlations of *UBE2I* expression patterns with tumor stages in pan-DSTs from the GEPIA database were further explored.

### 2.2. Survival and Immune Infiltrate Analyses in Pan-DSTs

Survival analyses, including overall survival (OS) and disease-free survival (DFS), of pan-DSTs from the GEPIA database were conducted. *UBE2I* expression in pan-DST samples was subdivided into either low or high group based on the median value. Next, we analyzed immune infiltrates in pan-DSTs in terms of gene expression, survival outcomes, and somatic copy number alterations (SCNAs) using the Tumor Immune Estimation Resource (TIMER; URL: https://cistrome.shinyapps.io/timer/) database [21, 22]. Specifically, the gene module mainly focused on the correlation of *UBE2I* expression with the abundance of immune infiltrates (B, CD4^+^ T, and CD8^+^ T cells, as well as neutrophils (NP), macrophages (MP) and dendritic cells (DC)), the survival module primarily discussed the correlation of survival outcomes with *UBE2I* expression and immune infiltrate abundance, and the SCNA module mainly investigated the correlation of somatic CNA with immune infiltrate abundance.

### 2.3. Promoter Methylation and Protein Expression Analyses in Pan-DSTs

Differential promoter methylation of *UBE2I* was evaluated by types as well as stratified analyses additionally conducted by gender, race and nodal metastasis in pan-DSTs. Subsequently, protein levels of *UBE2I* were analyzed by types and stratification of colon cancer by gender, race, tumor stage, and tumor histology (information on other pan-DSTs was not available from the database).

### 2.4. Interaction Networks Involving *UBE2I* in Pan-DSTs

The potential mechanisms underlying the prognostic significance of *UBE2I* in pan-DSTs were further explored. Related genes coexpressed with *UBE2I* in these tumors were identified from the cBioPortal database (URL: https://www.cbioportal.org/), and the top 100 were used for interaction network construction [23, 24]. Interaction networks of pathways (bioprocesses, cellular composition, molecular functions, immune processes, KEGG pathways, reactome pathways, and diseases, etc.) were generated with ClueGO plugin of Cytoscape software *v*3.7.2 [25, 26]. Gene-gene interaction (GGI) as well as protein-protein interaction (PPI) networks were constructed to explore potential interactions at gene and protein levels using geneMANIA (URL: http://genemania.org/) [27] and STRING (URL: https://string-db.org/) [28] databases, respectively.

### 2.5. Diagnostic Significance of UBE21 in Survival of DSTs

The diagnostic significance of *UBE2I* was determined based on the expression of *UBE2I* in pan-DSTs obtained from the Oncomine database (URL: https://www.oncomine.org/resource/main.html). Specifically, diagnostic significance was evaluated via receiver operating characteristic (ROC) curves constructed using both tumor and nontumor data. The criteria for the identification of potential diagnostic biomarkers were as follows: (1) those showing differential expression in tumor and nontumors and (2) those with an area under curve (AUC) ≥ 0.700 and a *P* ≤ 0.050. The Cancer Genome Atlas (TCGA) datasets, including COAD and PAAD, Alon colon cancer [29], and Logsdon pancreas [30] datasets, were used for evaluating the diagnostic value of *UBE2I*.

### 2.6. Statistical Analysis

One-way ANOVA was applied for gene expression analysis of *UBE2I* in different tumor stages. Analyses of differential expression patterns of *UBE2I* between carcinoma specimens and normal counterparts, as well as promoter methylation between groups, including differences between tumor and normal, male and female, different races, node metastasis and tumor grade categories, were performed via the Mann–Whitney *U* test. Survival analysis and the correlation of *UBE2I* expression with immune infiltrates were made via the log-rank test and the Spearman's correlation coefficients, respectively. The Cox proportional hazard ratio (HR) with a 95% confidence interval (95% CI) was calculated from the survival plots. *P* ≤ 0.05 indicated the presence of statistical significance.

## 3. Results

### 3.1. Differential *UBE2I* mRNA Expression in Pan-DSTs

**Figure 1:**
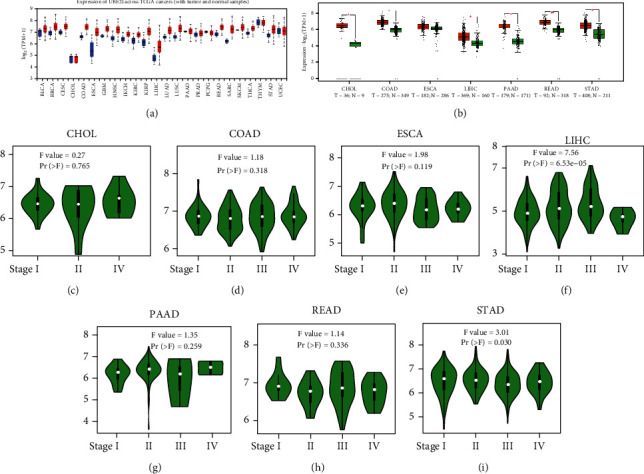
Differentialexpression and disease progression plots of *UBE2I* in pan-DSTs. (a) Differential expression patterns of *UBE2I* in pan-DSTs from TCGA. (b) Differential expression patterns of *UBE2I* in pan-DSTs. (c–i) Disease progression plots between *UBE2I* expression and tumor stage in CHOL, COAD, ESCA, LIHC, PAAD, READ, and STAD.

From the TCGA database, we obtained data of 7 different types of digestive system cancers, namely, cholangiocarcinoma (CHOL), colon adenocarcinoma (COAD), esophageal carcinoma (ESCA), liver hepatocellular carcinoma (LIHC), pancreatic adenocarcinoma (PAAD), rectal adenocarcinoma (READ), and stomach adenocarcinoma (STAD). Evaluation of *UBE2I* mRNA expression across TCGA cancers revealed upregulated *UBE2I* in carcinomas, versus normal counterparts, in most cases (Figure 1(a)). Except ESCA, differentially expressed *UBE2I* was observed across all other pan-DST types (all *P* ≤ 0.05, Figure 1(b)). Evaluation of expression by pan-DST staging showed that *UBE2I* was differentially expressed in the diver stage in LIHC and STAD (*P* < 0.0001, 0.030; Figures 1(f) and 1(i)) but not in other DST types (all *P* > 0.05, Figures 1(c)–1(e), 1(g), and 1(h)). Specifically, *UBE2I* expression was increased in stages I-III while decreased in stage IV in LIHC; however, the converse expression pattern was observed in STAD.

### 3.2. Survival Analysis of *UBE2I* in Pan-DSTs

**Figure 2:**
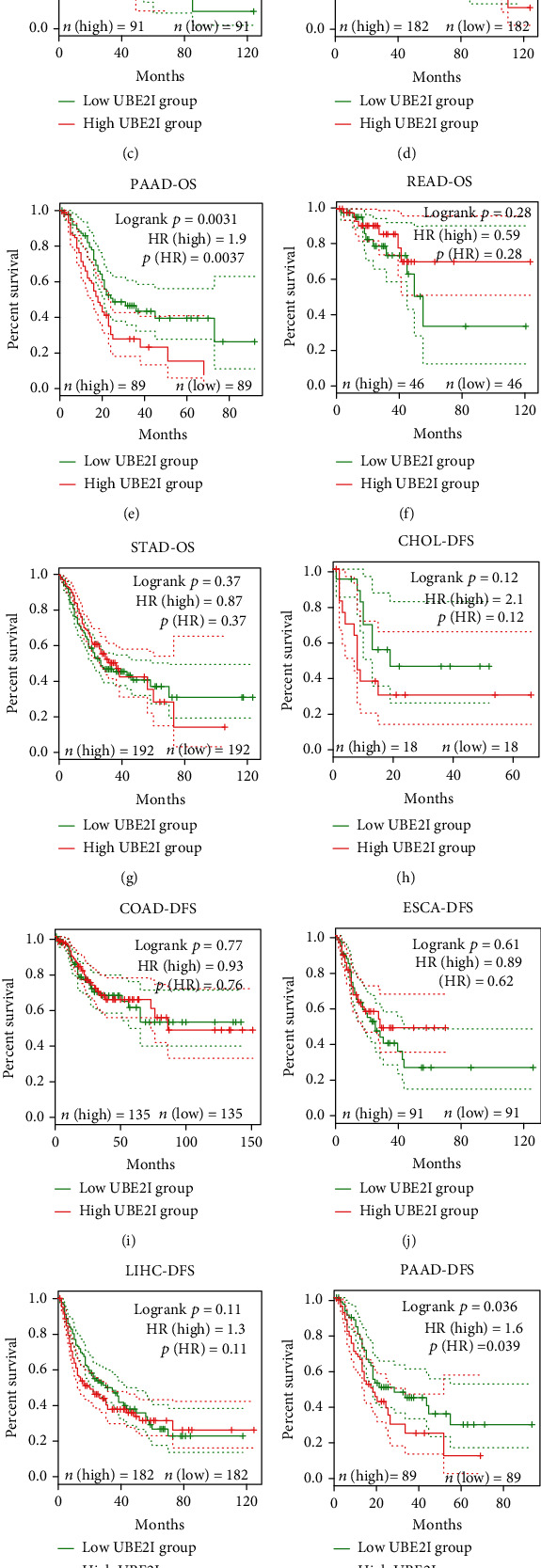
Overall and disease-free survival plots in relation to *UBE2I* expression in pan-DSTs. (a–g) Overall survival plots based on *UBE2I* expression in CHOL, COAD, ESCA, LIHC, PAAD, READ, and STAD. (h–n) Disease-free survival plots based on *UBE2I* expression in CHOL, COAD, ESCA, LIHC, PAAD, READ, and STAD.

Survival analyses, including OS and DFS, were carried out to determine the role of *UBE2I* expression in the prognosis of pan-DSTs. We observed favorable prognostic significance of *UBE2I* for OS in COAD and PAAD (log-rank [11] *P* = 0.049, HR (high) = 0.620; LR *P* = 0.003, HR (high) = 1.900; Figures 2(b) and 2(e)) but not in other DSTs examined (all LP *P* > 0.050; Figures 2(a), 2(c), 2(d), 2(f), and 2(g)). In terms of DFS, *UBE2I* showed favorable prognostic significance in PAAD only (LP *P* = 0.036, HR (high) = 1.600; Figure 2(l)). It suggests that upregulated *UBE2I* is beneficial for COAD but not for PAAD in terms of both OS and DFS.

### 3.3. Immune Infiltrate Analysis of UBE21 in Pan-DSTs

**Figure 3:**
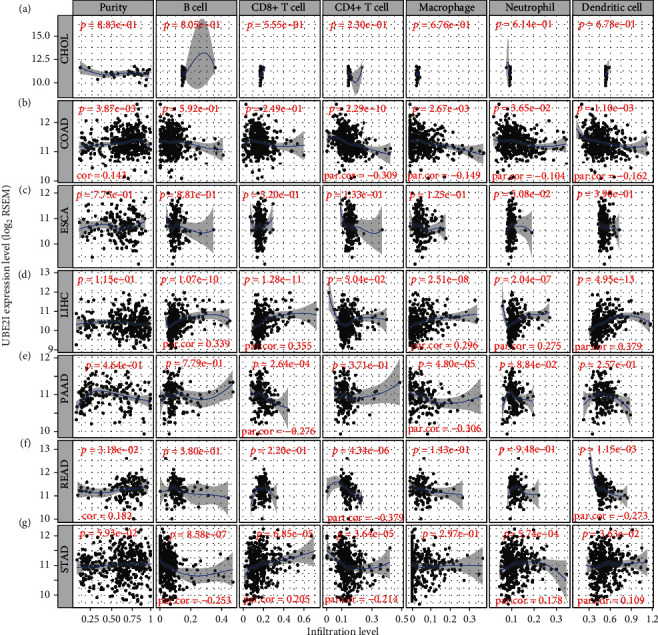
Correlation analysis of *UBE2I* expression with immune infiltrates. (a–g) Correlations of *UBE2I* with immune infiltrates (purity, B cells, CD8^+^ T cells, CD4^+^ T cells, MP, NP, and DC) in CHOL, COAD, ESCA, LIHC, PAAD, READ, and STAD.

Spearman's correlation coefficients were used to evaluate the correlation of *UBE2I* expression with immune infiltrates in a range of pan-DSTs. The data showed no connection between *UBE2I* expression and purity or immune infiltrates (B cells, CD4^+^, and CD8^+^ T cells, as well as NP, MP, and DC) in CHOL and ESCA (all *P* > 0.050, Figures 3(a) and 3(c)). In COAD, *UBE2I* was positively associated with purity but negatively with CD4^+^ T cells, MP, NP, and DC (all *P* < 0.050, *R* = 0.143, -0.309, -0.149, -0.104, and -0.162; Figure 3(b)). In LIHC, a positive association between *UBE2I* and B cells, CD8^+^ T cells, MP, NP, and DC was determined (all *P* < 0.050, *R* = 0.339, 0.355, 0.296, 0.275, and 0.379; Figure 3(d)). In PAAD, an inverse connection was found between *UBE2I* and CD8^+^ T cells and MP (both *P* < 0.050, *R* = −0.276 and -0.306; Figure 3(e)). In READ, *UBE2I* was positively linked to purity but negatively to CD4^+^ T and DC (all *P* < 0.050, *R* = 0.182, -0.37, and -0.273; Figure 3(f)). And in STAD, *UBE2I* was found to be positively correlated with CD8^+^ T cells, NP, and DC but had an inverse association with B and CD4^+^ T cells (all *P* < 0.050, *R* = 0.205, 0.178, 0.109, -0.253, and -0.214; Figure 3(g)).

**Figure 4:**
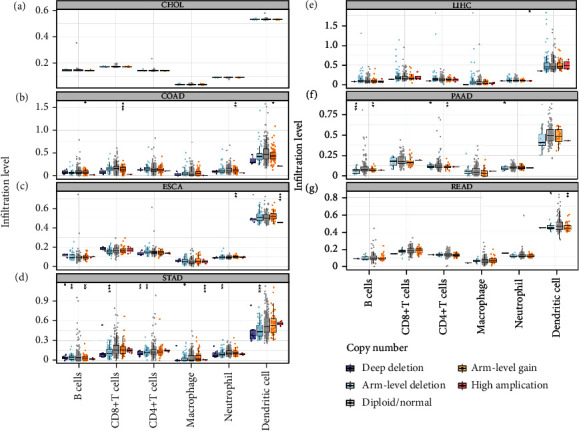
Analysis of associations between SCNAs of *UBE2I* and immune infiltrates of pan-DSTs. (a–g) Analysis of the correlation of SCNAs of *UBE2I* with immune infiltrates (B cells, CD8^+^ T cells, CD4^+^ T cells, MP NP, and DC) in CHOL, COAD, ESCA, LIHC, PAAD, READ, and STAD.

The potential connection between SCNAs of *UBE21* and immune infiltrates was further examined. Notably, no significant associations were observed between *UBE21* and all the six types of immune infiltrates (B cells, CD4^+^ T cells, CD8^+^ T cells, NP, MP, and DC) in CHOL (Figure 4(a)). In contrast, SCNAs of *UBE2I* (deep deletion, arm-level deletion and arm-level gain) were strongly linked to all the above six immune infiltrate types in STAD (Figure 4(g)). While in LIHC, SCNAs of *UBE2I* were significant only in relation to neutrophil amplification (Figure 4(d)). In READ, SCNAs of *UBE2I* (arm-level deletion and gain) showed statistical significance in relation to DC (Figure 4(f)). In COAD, SCNAs of *UBE2I* were strongly related to B cells, CD8^+^ T cells, NP, and DC in terms of arm-level gain (Figure 4(b)). In PAAD, a close connection between the arm-level deletion and gain of *UBE2I* and B cells, CD8^+^ T cells, and NP was determined (Figure 4(e)). In ESCA, arm-level gain of *UBE2I* showed a significant correlation with the high amplification of NP and DC (Figure 4(c)).

**Figure 5:**
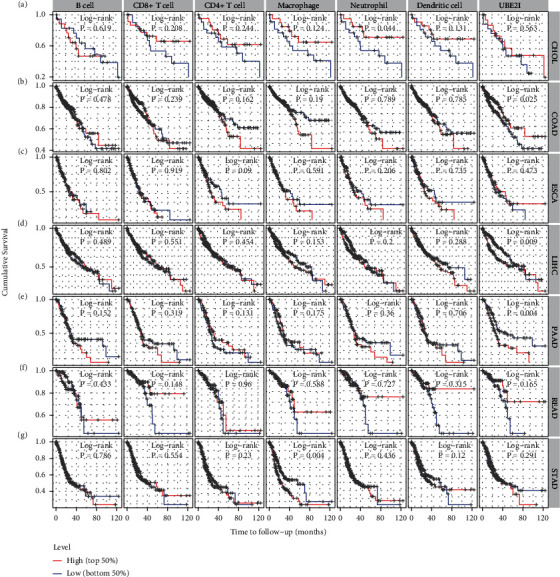
Immune infiltrate-related survival and *UBE2I* expression in pan-DSTs. (a–g) Immune infiltrate-related survival and *UBE2I* expression in CHOL, COAD, ESCA, LIHC, PAAD, READ, and STAD.

Next, prognostic analysis was performed based on immune infiltrates and *UBE2I* expression in pan-DSTs. The results showed that neutrophil infiltration was significantly correlated with CHOL while macrophage infiltration was correlated with STAD (log-rank *P* = 0.044, 0.004; Figures 5(a) and 5(g)). *UBE2I* expression showed favorable prognostic value in COAD, LIHC, and PAAD (log-rank *P* = 0.025, 0.009, and 0.004; Figures 5(b), 5(d), and 5(e)), but not in other cancer types.

### 3.4. Promoter Methylation Analysis of UBE2I in Pan-DSTs

**Figure 6:**
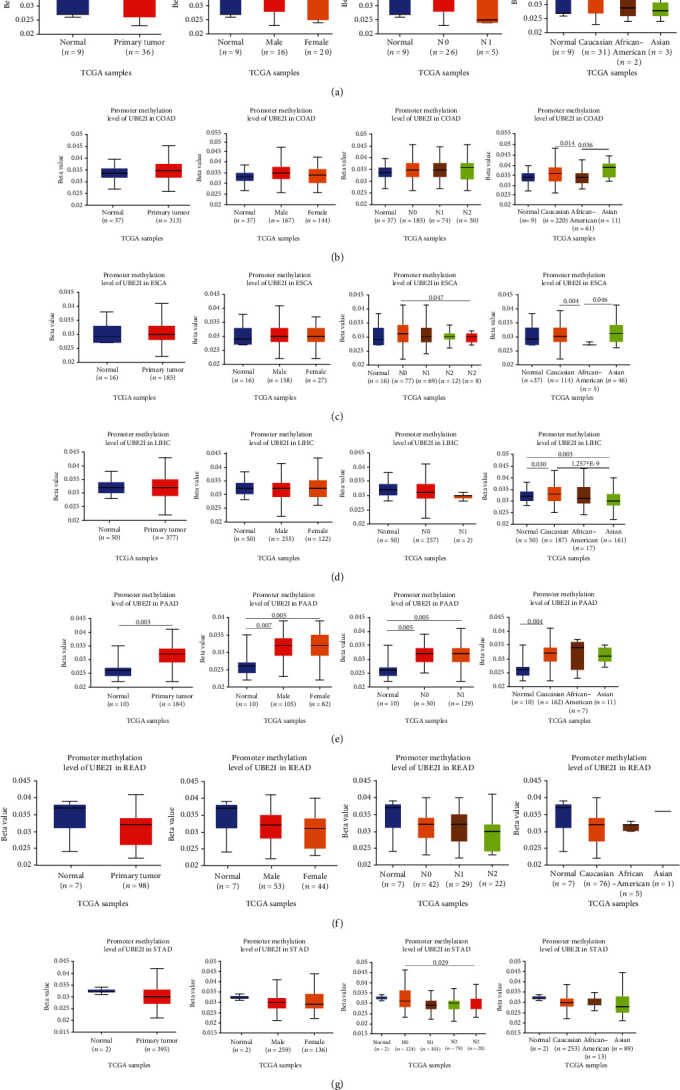
Promoter methylation analysis of *UBE2I* in pan-DSTs and stratified analyses by gender, lymph node, and race. (a–g) Promoter methylation analysis of *UBE2I* in pan-DSTs and stratified analyses by gender, lymph node, and race in CHOL, COAD, ESCA, LIHC, PAAD, READ, and STAD.

Promoter methylation analysis was initially applied to validate differential expression of *UBE2I* in tumor and normal samples. Differential methylation of *UBE2I* was observed in PAAD, with high methylation in tumor and low methylation in normal cells (*P* = 0.003; Figure 6(e)), but not in other DSTs (all *P* > 0.050; Figures 6(a)–6(d), 6(f), and 6(g)). Furthermore, upon stratification by gender, differential methylation of *UBE2I* was consistently observed in the PAAD subtype, with high methylation levels in both male and female populations, compared to their control counterparts (*P* = 0.007, 0.005; Figure 6(e)). Stratified analysis by nodal metastasis showed differential methylation of *UBE2I* in ESCA, PAAD, and STAD. Consistently, differential levels of methylated *UBE2I* were detected in nodal metastasis groups of ESCA and STAD, with high methylation at N0 and low methylation at N3 (*P* = 0.047, 0.029; Figures 6(c) and 6(g)). In PAAD, high methylated *UBE2I* levels were observed at both N0 and N1 stages, compared to normal samples (both *P* = 0.005; Figure 6(e)). Upon stratification by race, differential methylation of *UBE2I* was detected in COAD, ESCA, LIHC, and PAAD subtypes. Specifically, *UBE2I* methylation levels in COAD and ESCA were significantly higher in Asians and Caucasians than in African-Americans (*P* = 0.014, 0.036; 0.004, 0.046; Figures 6(b) and 6(c)). Higher methylation in Caucasians and lower methylation in Asians with LIHC were detected, compared to the corresponding control groups (*P* = 0.030, 0.003), with significant differences between the two races (*P* = 1.257∗*E* − 9; Figure 6(d)). The Caucasian subgroup of PAAD displayed high *UBE2I* methylation, compared to the corresponding control group (*P* = 0.004; Figure 6(e)).

### 3.5. Protein Expression Analysis of *UBE2I* in Colon Cancer

**Figure 7:**
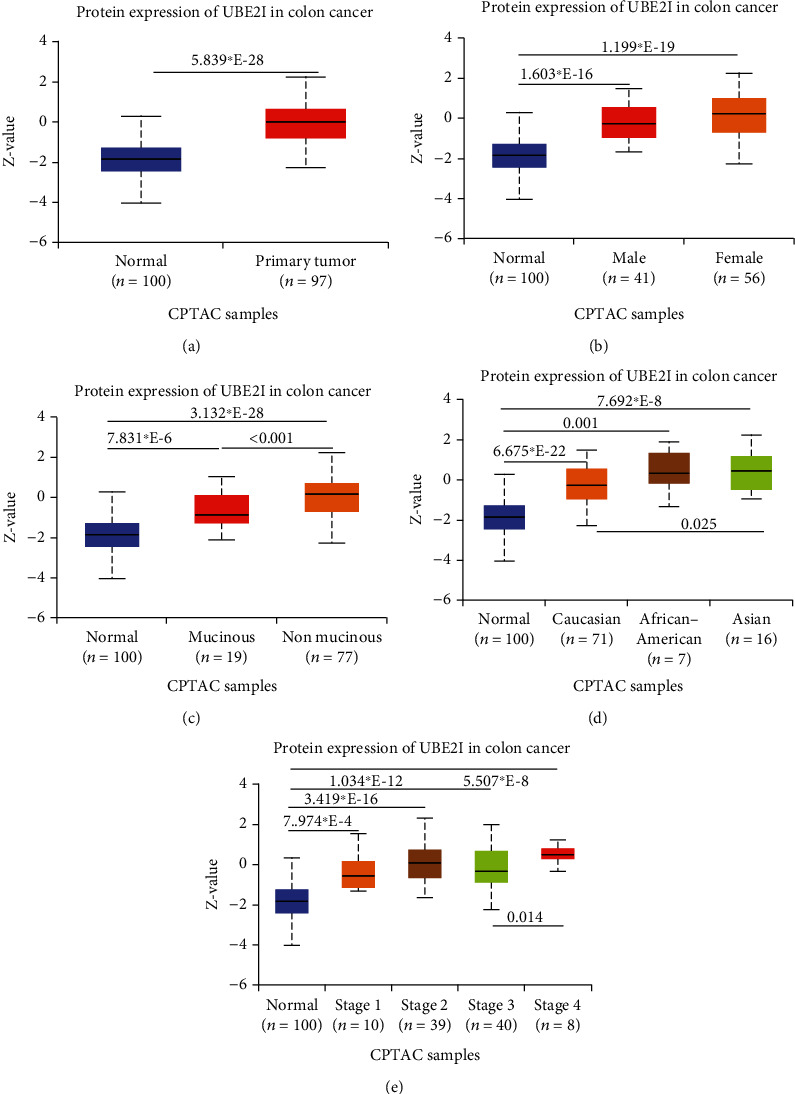
Protein expression analysis of *UBE2I* in tumors. (a–e) Stratified analyses by gender, race, tumor stage, and tumor histology in colon cancer.

*UBE2I* was highly expressed in primary colon tumors, versus normal counterparts (*P* = 5.839^∗^*E* − 28, Figure 7(a)). Stratified analyses by gender, tumor histology, race, and tumor staging consistently disclosed higher expression in tumor versus normal tissue. Both sexes in the tumor groups displayed higher UBE21 expression than their control counterparts (*P* = 1.603^∗^*E* − 16, 1.199^∗^*E* − 19; Figure 7(b)). High expression was detected in both mucinous and nonmucinous types, compared to normal tissue (*P* = 7.831^∗^*E* − 6, 3.132^∗^*E* − 28; Figure 7(c)), which was more marked in nonmucinous than mucinous tumors (*P* < 0.001). We also detected high expression in Caucasian, Franco-American, and Asian populations with colon cancer, compared to their control counterparts (*P* = 6.675^∗^*E* − 22, 0.001, 7.692^∗^*E* − 8; Figure 7(d)), with even higher expression in Asians versus Caucasians (*P* = 0.025). *UBE21* was upregulated in tumor stages I-IV (*P* = 7.974^∗^*E* − 4, 3.419^∗^*E* − 16, 1.034^∗^*E* − 12, and 5.507^∗^*E* − 8; Figure 7(e)), with higher expression in stage IV, compared to stage III carcinoma (*P* = 0.014).

### 3.6. Interaction Networks of Potential Pathways Involving *UBE2I* in COAD and PAAD

**Figure 8:**
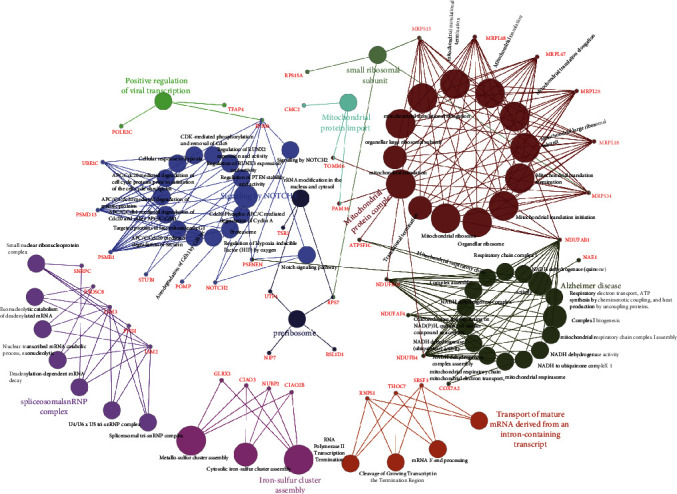
Illustration of functional enrichment pathways of *UBE2I* and coexpressed genes involved in COAD.

**Figure 9:**
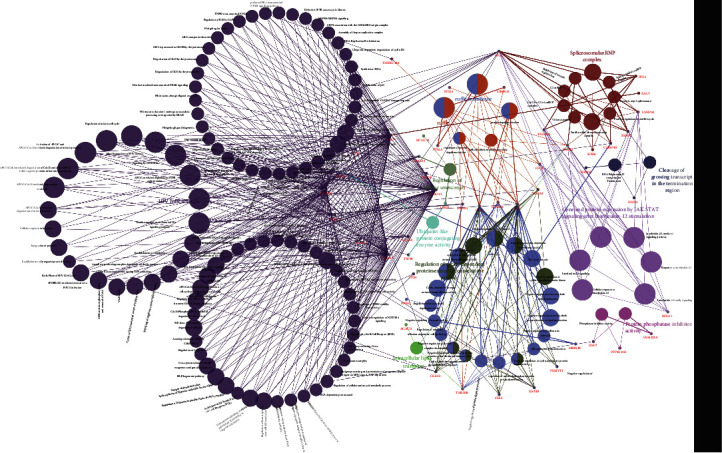
Illustration of interaction networks of *UBE2I* and coexpressed genes involved in PAAD.

**Figure 10:**
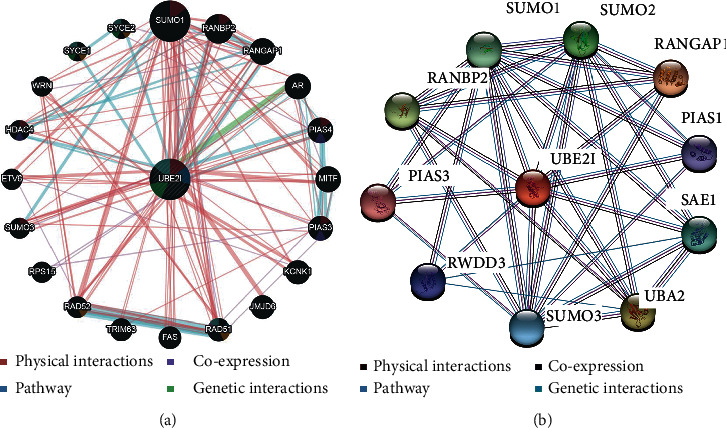
Gene-gene and protein-protein interaction networks. (a) Gene-gene interaction networks of *UBE2I* and related genes. (b) Protein-protein interaction networks of UBE2I and related proteins.

*UBE2I* coexpressed genes in the cBioPortal database were first identified for analysis. The top 100 genes related to *UBE2I* in COAD and PAAD are presented in Tables 1 and 2, respectively, based on which the interaction networks involving *UBE2I* in COAD were constructed (Figure 8). Associated genes were found to be involved in preribosome, transport of mature mRNAs derived from intron-containing transcripts, spliceosomal snRNP complexes, Notch signaling, mitochondrial protein complexes, small ribosomal subunits, and Alzheimer's disease. The interaction network of *UBE2I* in PAAD included genes involved in ruffle membrane, regulation of cellular senescence, spliceosomal snRNP complex, gene, and protein expression by JAK-STAT axis after interleukin-12 stimulation, protein phosphatase inhibitor activity, and HIV infection (Figure 9). Finally, GGI and PPI networks were constructed to visualize these relationships. GGI analysis showed physical, coexpression, pathway, and genetic interactions of *UBE21* with *SUMO1*, *SUMO3*, *RANBP2*, *SYCE2*, *SYCE1*, *PIAS3*, *PIAS4*, *RAD51*, and *RAD52* (Figure 10(a)). In PPI analysis, physical, coexpression, pathway, and genetic interactions of UBE21 with SUMO1-3, SAE1, PIAS1, PIAS3, UBA2, and RWDD3 were detected (Figure 10(b)).

### 3.7. Diagnostic Value of UBE2I in Pan-DSTs

**Figure 11:**
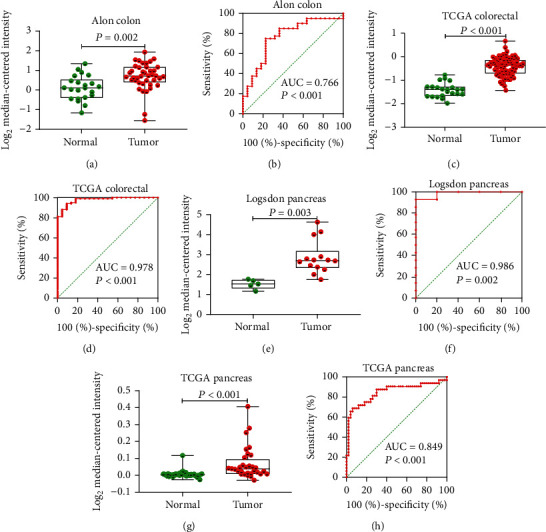
Differential expression and diagnostic ROC curves of *UBE2I* in COAD and PAAD. (a, c) Differential expression of *UBE2I* in COAD. (b, d) Diagnostic ROC curve of *UBE2I* in COAD. (e, g) Differential expression of *UBE2I* in PAAD. (f, h) Diagnostic ROC curve of *UBE2I* in PAAD.

In view of the prognostic significance of *UBE2I* in COAD and PAAD, its diagnostic value in these cancer types was further explored. *UBE2I* displayed differential expression and favorable diagnostic value in COAD of TCGA colorectal and Alon datasets (AUC = 0.766 and 0.978, *P* = 0.002, <0.001, <0.001, and <0.001; Figures 11(a)–11(d)). Moreover, *UBE2I* showed differential expression and good diagnostic value in PAAD of TCGA pancreas and Logsdon datasets (AUC = 0.986 and 0.849, *P* = 0.003, 0.002, <0.001, and <0.001; Figures 11(e)–11(h)).

## 4. Discussion

The current research explored the potential correlation of *UBE2I* expression with a range of pan-DSTs, including CHOL, COAD, ESCA, LIHC, PAAD, READ, and STAD. Our data preliminary revealed differential expression of *UBE2I*, with higher expression in all tumor types, except ESCA. Interestingly, survival analysis indicated that high *UBE2I* expression was associated with adverse OS and DFS in PAAD but improved OS in READ. In immune infiltrate analysis, *UBE2I* expression was partially associated with purity or B cells, CD8^+^ and CD4^+^ T cells, MP, NP, and DC in COAD, LIHC, PAAD, READ, and STAD. Evaluation of the correlation between SCNAs and immune infiltrates revealed that *UBE2I* was associated with all six immune infiltrate types in STAD but partially associated with specific immune cell types in the other five pan-DST types. Differential promoter methylation of *UBE2I* was observed in PAAD only, with high methylation in tumor and low methylation in normal tissues. Consistently, stratified analyses by gender, nodal metastasis, and race showed differential methylation in PAAD, which was also partially observed in COAD, ESCA, and STAD. In colon cancer, differential UBE2I protein expression was observed as well as following stratification by gender, tumor histology, race, and tumor stage. Analysis of the interaction networks of potential pathways disclosed involvement of *UBE2I* in the spliceosomal snRNP complex, Notch signaling pathway, mitochondrial protein complex, small ribosomal subunit, Alzheimer's disease, protein phosphatase inhibitor activity, and HIV infection in COAD and PAAD. Furthermore, *UBE2I* showed differential expression and favorable diagnostic value in COAD and PAAD from two separate datasets.

Digestive system carcinomas comprise many types of neoplasms including esophageal cancer, gastric cancer, small and large bowel cancers, and cancers from liver and bile duct system, pancreas, and anal regions [31], which are reported to contribute to more than 3.2 billion deaths worldwide [2]. In addition, digestive system malignancies account for ~40% of cancer-related deaths worldwide with substantial adverse effects on both developed and developing countries [4]. Thus, identifying novel biomarkers for early diagnosis and survival surveillance for high-risk populations and postsurgical patients remains an urgent medical requirement. Digestive system carcinoma subtypes in the TCGA database include CHOL, ESCA, LIHC, PAAD, COAD, READ, and STAD. To our knowledge, no studies to date have determined the expression profiles and clinical implications of *UBE2I*, also known as *UBC9*, in these pan-DSTs.

*UBC9*, the unique E2-conjugating enzyme needed for SUMOylation, is a core modulator of essential cellular functions and changes frequently in cancer, contributing substantially to the progression of human tumors [32]. Mattoscio and Chiocca [33] previously suggested that upregulation of *UBC9* in HGSOC cells with *BRCA1* mutations leads to loss of caveolin-1 and induction of vascular epithelial growth factor, supporting a pathway linking *BRCA1* mutation in HGSOC with peritoneal permeability and ascites formation. The same group reported that knockdown of *UBC9* in BRCA1 mutant triple-negative breast cancer and HGSOC cells inhibited cell proliferation and migration, indicating the pivotal role of *UBC9* in endothelial-mesenchymal transition in such cancer type [34]. Epithelial-mesenchymal transition is a biological phenomenon whereby epithelial cells show enhanced migration ability to distal sites, facilitating tumor metastasis [35]. These findings support critical roles of *UBC9* expression and dependent pathways in metastasis of triple-negative breast cancer. Similarly, our results suggest association of high *UBC9* expression with poor prognosis in PAAD as well as differential levels (low in normal samples) of promoter methylation concerning lymph node metastasis. The role of *UBC9* in PAAD in our study is consistent with that reported in other studies across diverse tumor types, including lung, colorectal, prostate, ovarian, and breast cancers as well as melanoma [36–41]. In addition, we determined the diagnostic potential of UBE2I in PAAD and COAD, which has rarely been reported in cancers. Our data showed favorable prognostic value of *UBE2I* in COAD but not in PAAD. A previous study on 602 early-stage colorectal cancer patients by Fridman et al. [42] revealed the presence of high memory T-cell (CD45RO^+^ and CD8^+^) infiltrates in tumors. In our experiments, *UBE2I* expression was positively correlated with purity and inversely with CD4^+^ T cells, MP, NP, and DC rather than CD8^+^ T cells, regardless of tumor stages. However, whether this inconsistency is associated with tumor staging or other influencing factors is yet to be elucidated.

Promoter methylation is the most extensively characterized type of epigenetic alteration; in particular, DNA methylation in CpG islands is predominantly present in the upstream promoter region that takes responsibility for inhibitory protein complex recruitment, inducing transcriptional repression of downstream genes [43]. There was once a proposal that DNA methylation alterations may contribute to oncogenesis, as the cytosine base of DNA was initially found to be methylated to 5-methylcytosine, or the fifth base [44]. Recent evidence has shown that 5-methylcytosine distribution alterations can help effectively differentiate cancer from normal cells, with focal hypermethylation of tumor suppressor gene promoters identified as one of the main mechanisms [44]. Homeostasis alterations of epigenetic mechanisms are crucial to the development of human cancers [44]. Consistently, our experiments showed increased promoter hypermethylation of *UBE2I* in PAAD tumors relative to control, which retained significance upon stratification by gender, race, and nodal metastasis, supporting the involvement of *UBE2I* hypermethylation in the development of PAAD. However, no data on methylation levels and prognostic significance on *UBE2I* were available for other pan-DST types.

Marked advances in tumor immunotherapy have been attributed to the increasing awareness of the importance of the tumor immune microenvironment in inhibiting antitumor immunity [45]. Overcoming the ability of cancer cells to evade immune detection allows the available treatment approaches for multiple cancer types to attack tumors via harnessing the “non-self”-directed specificity of the immune system [45]. One of the most promising therapeutic strategies for antitumor immunity reactivation is pharmacological manipulation of physiological immune checkpoints [45]. Exploiting immune checkpoint pathways is a major mechanism for tumors to escape immune surveillance, so immune checkpoint blockade underlies the antineoplasmic activity of most approved agents targeting CTLA-4, as well as programmed cell death protein-1/ligand-1 [46]. Additionally, a number of predictive biomarkers, such as abundance and location of tumor-infiltrating lymphocytes, have been explored for immune-oncology applications [42]. Established findings suggest that local inflammation significantly affects tumor progression. The group further showed that highly adaptive immune infiltrates of intratumoral lymphocytes present a crucial prognostic marker for solid tumors [42].

Solid tumors are often infiltrated by immune cells, including T and B lymphocytes, natural killer cells, DC, MP, NP, eosinophils, and mast cells [42]. Dunn and coworkers reported an association of immune deficiency with tumor proliferation and aggressiveness in a mouse model [47]. Clinical, experimental and epidemiological studies have indicated chronic inflammation as an important inducer of various cancer types [48], such as *Helicobacter pylori* infection in gastric cancer [49] and mucosal lymphoma [50]. The presence of lymphocytes in large quantities, especially T cells, in contrast to infiltration of cells responsible for chronic inflammation, is considered a beneficial prognostic marker for diverse cancer types, including melanoma, non-Hodgkin's lymphoma, head-and-neck cancer, non-small-cell lung cancer, and breast, ovarian, esophageal, and urothelial carcinomas [42, 51–53]. Data from the current study indicate that *UBE2I* expression is associated with six immune infiltrate cell types and purity in COAD, LIHC, PAAD, READ, and STAD. Similar to earlier findings, a high NP count was associated with favorable prognosis in CHOL while a high MP count indicated adverse prognosis in STAD. Further research is warranted to explain this differential prognostic relevance.

This study has a number of limitations that should be taken into consideration. First, the findings obtained require further validation in other cohorts on a larger scale. Second, it is essential to clarify the mechanisms of *UBE2I* in COAD and PAAD *in vivo* and *in vitro*. Third, clinical translation needs to be explored for optimizing therapeutic application.

## Figures and Tables

**Table 1:** Coexpression-related genes of *UBE2I* in COAD.

Correlated gene	Cytoband	Spearman's correlation	*P* value	*q* value
RNPS1	16p13.3	0.652	4.92*E* − 73	9.85*E* − 69
NDUFAB1	16p12.2	0.639	2.76*E* − 69	2.77*E* − 65
JPT2	16p13.3	0.626	7.87*E* − 66	5.25*E* − 62
PAM16	16p13.3	0.552	1.81*E* − 48	9.05*E* − 45
C16ORF91	16p13.3	0.542	1.40*E* − 46	5.59*E* − 43
NIP7	16q22.1	0.531	2.23*E* − 44	7.45*E* − 41
FOPNL	16p13.11	0.518	5.71*E* − 42	1.63*E* − 38
HSBP1	16q23.3	0.516	1.35*E* − 41	3.37*E* − 38
DNAJA2	16q11.2	0.516	1.52*E* − 41	3.39*E* − 38
KCTD5	16p13.3	0.512	6.05*E* − 41	1.21*E* − 37
PMM2	16p13.2	0.509	2.88*E* − 40	4.81*E* − 37
LYRM1	16p12.3	0.509	2.88*E* − 40	4.81*E* − 37
DNAJA3	16p13.3	0.507	6.38*E* − 40	9.82*E* − 37
CFAP20	16q21	0.506	7.81*E* − 40	1.12*E* − 36
KARS	16q23.1	0.5	1.07*E* − 38	1.43*E* − 35
RSL1D1	16p13.13	0.498	1.95*E* − 38	2.44*E* − 35
NDUFB4	3q13.33	0.496	4.52*E* − 38	5.32*E* − 35
GINS2	16q24.1	0.495	5.90*E* − 38	6.57*E* − 35
NUTF2	16q22.1	0.493	1.58*E* − 37	1.67*E* − 34
MRPS34	16p13.3	0.491	3.54*E* − 37	3.54*E* − 34
NUBP2	16p13.3	0.488	1.07*E* − 36	1.02*E* − 33
POMP	13q12.3	0.487	1.52*E* − 36	1.39*E* − 33
TOMM6	6p21.1	0.484	5.17*E* − 36	4.50*E* − 33
SNRPC	6p21.31	0.483	6.16*E* − 36	5.14*E* − 33
PSMB1	6q27	0.48	1.68*E* − 35	1.35*E* − 32
EEF2KMT	16p13.3	0.475	1.24*E* − 34	9.55*E* − 32
EXOSC8	13q13.3	0.473	2.57*E* − 34	1.91*E* − 31
NAE1	16q22.1	0.471	4.38*E* − 34	3.13*E* − 31
EP300	22q13.2	-0.47	7.31*E* − 34	5.05*E* − 31
HEBP2	6q24.1	0.467	2.00*E* − 33	1.33*E* − 30
METTL9	16p12.2	0.467	2.09*E* − 33	1.33*E* − 30
CMC2	16q23.2	0.467	2.13*E* − 33	1.33*E* − 30
MSRB1	16p13.3	0.465	4.67*E* − 33	2.83*E* − 30
CENPN	16q23.2	0.465	5.04*E* − 33	2.97*E* − 30
METTL26	16p13.3	0.464	5.65*E* − 33	3.23*E* − 30
CLIC1	6p21.33	0.463	9.56*E* − 33	5.32*E* − 30
MRPL18	6q25.3	0.462	1.03*E* − 32	5.59*E* − 30
RPS15A	16p12.3	0.459	4.01*E* − 32	2.11*E* − 29
EMC8	16q24.1	0.457	8.05*E* − 32	4.13*E* − 29
HERC1	15q22.31	-0.454	1.65*E* − 31	8.28*E* − 29
NDUFB10	16p13.3	0.454	2.01*E* − 31	9.84*E* − 29
THOC7	3p14.1	0.454	2.13*E* − 31	1.02*E* − 28
CIAO2B	16q22.1	0.452	3.81*E* − 31	1.77*E* − 28
CYB5B	16q22.1	0.451	4.67*E* − 31	2.12*E* − 28
TSR3	16p13.3	0.451	5.35*E* − 31	2.38*E* − 28
NOTCH2	1p12	-0.45	6.77*E* − 31	2.95*E* − 28
FAM192A	16q13	0.45	7.97*E* − 31	3.39*E* − 28
MTMR3	22q12.2	-0.45	8.43*E* − 31	3.52*E* − 28
PSMD13	11p15.5	0.449	8.87*E* − 31	3.62*E* − 28
NBPF10	1q21.1	-0.449	1.12*E* − 30	4.47*E* − 28
UCHL3	13q22.2	0.449	1.17*E* − 30	4.61*E* − 28
POLR2C	16q21	0.448	1.70*E* − 30	6.56*E* − 28
TMEM208	16q22.1	0.447	2.03*E* − 30	7.66*E* − 28
MRPS15	1p34.3	0.446	2.62*E* − 30	9.72*E* − 28
HMGB1	13q12.3	0.446	3.08*E* − 30	1.12*E* − 27
DIP2A	21q22.3	-0.444	5.29*E* − 30	1.89*E* − 27
ZSWIM8	10q22.2	-0.444	6.15*E* − 30	2.16*E* − 27
NDUFAF4	6q16.1	0.443	7.55*E* − 30	2.61*E* − 27
ARPC4	3p25.3	0.442	9.40*E* − 30	3.19*E* − 27
PI4KA	22q11.21	-0.442	1.02*E* − 29	3.39*E* − 27
MRPL47	3q26.33	0.442	1.12*E* − 29	3.69*E* − 27
ZNF263	16p13.3	0.441	1.67*E* − 29	5.40*E* − 27
STUB1	16p13.3	0.44	1.81*E* − 29	5.75*E* − 27
RPUSD1	16p13.3	0.44	2.17*E* − 29	6.69*E* − 27
FAM168A	11q13.4	-0.44	2.17*E* − 29	6.69*E* − 27
UBE2C	20q13.12	0.439	2.65*E* − 29	8.04*E* − 27
ZFYVE1	14q24.2	-0.438	3.28*E* − 29	9.80*E* − 27
SRSF3	6p21.31-p21.2	0.438	3.62*E* − 29	1.07*E* − 26
PRDX1	1p34.1	0.437	4.54*E* − 29	1.32*E* − 26
PSENEN	19q13.12	0.436	6.31*E* − 29	1.80*E* − 26
BCL7C	16p11.2	0.436	7.23*E* − 29	2.04*E* − 26
TMEM186	16p13.2	0.434	1.56*E* − 28	4.35*E* − 26
UTP4	16q22.1	0.434	1.61*E* − 28	4.42*E* − 26
PPIH	1p34.2	0.433	1.84*E* − 28	4.97*E* − 26
BFAR	16p13.12	0.432	2.31*E* − 28	6.17*E* − 26
LSM2	6p21.33	0.431	3.36*E* − 28	8.86*E* − 26
GLRX3	10q26.3	0.431	3.72*E* − 28	9.67*E* − 26
BANF1	11q13.1	0.429	5.85*E* − 28	1.50*E* − 25
BCL2L2	14q11.2	-0.429	6.01*E* − 28	1.52*E* − 25
COX7A2	6q14.1	0.429	6.73*E* − 28	1.68*E* − 25
MRPL28	16p13.3	0.429	7.08*E* − 28	1.75*E* − 25
DCTPP1	16p11.2	0.429	7.54*E* − 28	1.84*E* − 25
PTMA	2q37.1	0.427	1.44*E* − 27	3.48*E* − 25
ATP5F1C	10p14	0.426	1.58*E* − 27	3.78*E* − 25
DYNC1H1	14q32.31	-0.425	2.13*E* − 27	5.02*E* − 25
RPS7	2p25.3	0.425	2.24*E* − 27	5.21*E* − 25
MCRIP2	16p13.3	0.424	3.50*E* − 27	8.07*E* − 25
ZNF236	18q23	-0.423	3.76*E* − 27	8.56*E* − 25
TFAP4	16p13.3	0.422	5.43*E* − 27	1.22*E* − 24
AKAP13	15q25.3	-0.422	5.96*E* − 27	1.33*E* − 24
ZFYVE26	14q24.1	-0.421	7.79*E* − 27	1.71*E* − 24
GOT2	16q21	0.421	8.77*E* − 27	1.91*E* − 24
COPS9	2q37.3	0.42	1.19*E* − 26	2.57*E* − 24
TECPR2	14q32.31	-0.419	1.41*E* − 26	3.00*E* − 24
HECTD4	12q24.13	-0.419	1.67*E* − 26	3.52*E* − 24
CIAO3	16p13.3	0.418	1.69*E* − 26	3.52*E* − 24
FYCO1	3p21.31	-0.418	1.76*E* − 26	3.63*E* − 24
MRPL48	11q13.4	0.418	1.97*E* − 26	4.02*E* − 24
LSM3	3p25.1	0.417	2.53*E* − 26	5.12*E* − 24
ANKRD52	12q13.3	-0.417	2.58*E* − 26	5.16*E* − 24

**Table 2:** Coexpression-related genes of UBE2I in PAAD.

Correlated gene	Cytoband	Spearman's correlation	*P* value	*q* value
TMSB10	2p11.2	0.75	3.44*E* − 33	6.87*E* − 29
PTMA	2q37.1	0.678	3.91*E* − 25	2.75*E* − 21
EIF6	20q11.22	0.677	4.13*E* − 25	2.75*E* − 21
PPIA	7p13	0.673	1.13*E* − 24	5.10*E* − 21
MFSD2B	2p23.3	0.672	1.28*E* − 24	5.10*E* − 21
PFDN2	1q23.3	0.671	1.53*E* − 24	5.10*E* − 21
SNRPA1	15q26.3	0.671	1.82*E* − 24	5.20*E* − 21
PPP4C	16p11.2	0.664	6.75*E* − 24	1.69*E* − 20
NUTF2	16q22.1	0.66	1.71*E* − 23	3.80*E* − 20
CFL1	11q13.1	0.657	3.06*E* − 23	6.12*E* − 20
RNF181	2p11.2	0.653	7.23*E* − 23	1.31*E* − 19
PPIH	1p34.2	0.652	9.20*E* − 23	1.53*E* − 19
RNPS1	16p13.3	0.649	1.61*E* − 22	2.48*E* − 19
PSMD13	11p15.5	0.638	1.20*E* − 21	1.71*E* − 18
S100A16	1q21.3	0.638	1.35*E* − 21	1.80*E* − 18
BANF1	11q13.1	0.634	2.74*E* − 21	3.43*E* − 18
KANK1	9p24.3	-0.633	3.12*E* − 21	3.67*E* − 18
CLASP2	3p22.3	-0.632	3.82*E* − 21	4.02*E* − 18
NOP10	15q14	0.632	3.82*E* − 21	4.02*E* − 18
S100A11	1q21.3	0.629	6.69*E* − 21	6.68*E* − 18
CKS1B	1q21.3	0.626	1.19*E* − 20	1.13*E* − 17
HMGA1	6p21.31	0.626	1.24*E* − 20	1.13*E* − 17
BOLA2	16p11.2	0.62	3.42*E* − 20	2.97*E* − 17
NUDT1	7p22.3	0.619	4.09*E* − 20	3.41*E* − 17
PPP1R14B	11q13.1	0.618	4.66*E* − 20	3.73*E* − 17
MRGBP	20q13.33	0.618	5.15*E* − 20	3.96*E* − 17
SNRPG	2p13.3	0.617	5.81*E* − 20	4.30*E* − 17
MBLAC2	5q14.3	-0.617	6.18*E* − 20	4.41*E* − 17
RALY	20q11.22	0.617	6.40*E* − 20	4.41*E* − 17
KCTD5	16p13.3	0.616	6.81*E* − 20	4.54*E* − 17
NCOA1	2p23.3	-0.611	1.57*E* − 19	9.87*E* − 17
S100A10	1q21.3	0.611	1.58*E* − 19	9.87*E* − 17
UBE2C	20q13.12	0.61	1.87*E* − 19	1.13*E* − 16
ZNF420	19q13.12	-0.61	2.01*E* − 19	1.18*E* − 16
ICA1L	2q33.2	-0.61	2.12*E* − 19	1.21*E* − 16
FNDC3A	13q14.2	-0.609	2.44*E* − 19	1.36*E* − 16
ELOB	16p13.3	0.608	3.02*E* − 19	1.63*E* − 16
CHMP4B	20q11.22	0.606	3.92*E* − 19	2.06*E* − 16
METTL7A	12q13.12	-0.605	4.38*E* − 19	2.25*E* − 16
C16ORF91	16p13.3	0.605	4.53*E* − 19	2.26*E* − 16
CPEB4	5q35.2	-0.604	5.29*E* − 19	2.58*E* − 16
STX4	16p11.2	0.604	5.56*E* − 19	2.65*E* − 16
TMEM189	20q13.13	0.602	7.50*E* − 19	3.48*E* − 16
SYNJ1	21q22.11	-0.602	8.40*E* − 19	3.82*E* − 16
CKS2	9q22.2	0.599	1.33*E* − 18	5.87*E* − 16
CIB1	15q26.1	0.599	1.35*E* − 18	5.87*E* − 16
ZNF471	19q13.43	-0.597	1.70*E* − 18	7.21*E* − 16
FAM122A	9q21.11	-0.597	1.91*E* − 18	7.95*E* − 16
AMIGO1	1p13.3	-0.596	2.13*E* − 18	8.69*E* − 16
PTTG1	5q33.3	0.595	2.32*E* − 18	9.28*E* − 16
ZNF37A	10p11.1	-0.595	2.58*E* − 18	9.93*E* − 16
SMARCA2	9p24.3	-0.595	2.58*E* − 18	9.93*E* − 16
PSMA7	20q13.33	0.594	2.83*E* − 18	1.07*E* − 15
THOC6	16p13.3	0.593	3.49*E* − 18	1.29*E* − 15
TNFRSF12A	16p13.3	0.592	3.73*E* − 18	1.34*E* − 15
RNF7	3q23	0.592	3.74*E* − 18	1.34*E* − 15
RNF180	5q12.3	-0.591	4.38*E* − 18	1.54*E* − 15
WASF3	13q12.13	-0.59	5.81*E* − 18	1.98*E* − 15
SCNM1	1q21.3	0.59	5.84*E* − 18	1.98*E* − 15
PSMD4	1q21.3	0.589	6.07*E* − 18	2.02*E* − 15
PSMA1	11p15.2	0.589	6.15*E* − 18	2.02*E* − 15
BCL2L12	19q13.33	0.588	7.19*E* − 18	2.32*E* − 15
MRPL28	16p13.3	0.588	7.80*E* − 18	2.48*E* − 15
REV3L	6q21	-0.587	8.64*E* − 18	2.70*E* − 15
NBEA	13q13.3	-0.586	9.74*E* − 18	3.00*E* − 15
TSTD2	9q22.33	-0.586	9.96*E* − 18	3.02*E* − 15
HCFC2	12q23.3	-0.586	1.13*E* − 17	3.37*E* − 15
ACADSB	10q26.13	-0.585	1.22*E* − 17	3.59*E* − 15
SETBP1	18q12.3	-0.585	1.30*E* − 17	3.78*E* − 15
C19ORF33	19q13.2	0.583	1.64*E* − 17	4.68*E* − 15
ZNF429	19p12	-0.583	1.72*E* − 17	4.79*E* − 15
ACACB	12q24.11	-0.583	1.73*E* − 17	4.79*E* − 15
PKMYT1	16p13.3	0.583	1.77*E* − 17	4.85*E* − 15
ANXA2	15q22.2	0.582	1.83*E* − 17	4.94*E* − 15
EIF3M	11p13	0.582	1.94*E* − 17	5.17*E* − 15
FTSJ1	Xp11.23	0.582	2.09*E* − 17	5.48*E* − 15
KAT2B	3p24.3	-0.581	2.32*E* − 17	6.01*E* − 15
CTTNBP2	7q31.31	-0.58	2.78*E* − 17	7.12*E* − 15
SLC2A13	12q12	-0.58	2.86*E* − 17	7.23*E* − 15
LONRF2	2q11.2	-0.579	2.98*E* − 17	7.35*E* − 15
ANKFY1	17p13.2	-0.579	2.98*E* − 17	7.35*E* − 15
TAF10	11p15.4	0.579	3.08*E* − 17	7.50*E* − 15
ADAMTSL3	15q25.2	-0.578	3.51*E* − 17	8.41*E* − 15
MRPS6	21q22.11	0.578	3.53*E* − 17	8.41*E* − 15
MAML3	4q31.1	-0.578	3.69*E* − 17	8.67*E* − 15
ZBED3	5q13.3	-0.576	4.61*E* − 17	1.06*E* − 14
LEMD1	1q32.1	0.576	4.62*E* − 17	1.06*E* − 14
TRMT112	11q13.1	0.576	4.81*E* − 17	1.09*E* − 14
PEG3	19q13.43	-0.576	4.92*E* − 17	1.10*E* − 14
OST4	2p23.3	0.576	4.93*E* − 17	1.10*E* − 14
APC	5q22.2	-0.575	5.37*E* − 17	1.17*E* − 14
LYPLA2	1p36.11	0.575	5.38*E* − 17	1.17*E* − 14
SF3B6	2p23.3	0.575	5.72*E* − 17	1.23*E* − 14
KLHDC1	14q21.3	-0.575	5.91*E* − 17	1.26*E* − 14
SELENOH	11q12.1	0.575	6.09*E* − 17	1.28*E* − 14
ULK2	17p11.2	-0.574	6.44*E* − 17	1.34*E* − 14
TALDO1	11p15.5	0.574	6.61*E* − 17	1.36*E* − 14
PREPL	2p21	-0.574	6.70*E* − 17	1.37*E* − 14
APPBP2	17q23.2	-0.574	6.80*E* − 17	1.37*E* − 14
KCNJ3	2q24.1	-0.573	7.98*E* − 17	1.59*E* − 14

## Data Availability

The authors confirm that the data supporting the findings of this study are available within the article.
